# Role of the light fraction of soil organic matter in trace elements binding

**DOI:** 10.1371/journal.pone.0217077

**Published:** 2019-05-30

**Authors:** Katarzyna Wiatrowska, Jolanta Komisarek

**Affiliations:** Departament of Soil Science and Land Reclamation, Poznan University of Life Sciences, Poznań, Poland; University of Vigo, SPAIN

## Abstract

The light fraction of soil organic matter (LF) has a rapid turnover and may be potentially metal-enriched, but the interaction between this pool of organic matter and trace elements has not been well studied. The present study aimed to investigate changes in LF content and its effect on heavy metals distribution and extractability in long-term contaminated soil by smelting activity. An incubation experiment was conducted on a surface horizon of Brunic Arenosol sampled from the previously-existing sanitary zone of Głogów smelter, for 450 days. The contaminated soil was divided into three combinations: with the addition of either triticate straw (at the dose of 4.5 Mg ha^-1^) or pig manure (at the dose of 40 Mg ha^-1^) or without any “foreign” organic materials (nil). The LF (ρ > 1.7 g cm^-3^) occurred to be metal-enriched and despite its low content (5.49%—nil, 7.18%—straw and 7.29%—manure combination) in the bulk soil, it was observed that initially Cd, Cu, Pb and Zn stock reached 16.2%, 11.9%, 18.0% and 32.3%, respectively. Incubation conditions where mineralization processes dominate led to a decrease in the LF share by about 12.6% in nil and 31.4–39.8% in the combinations with organic amendments. In consequence, the DOC (dissolved organic carbon) concentration doubled and metal distribution had changed. The increase in water-soluble (F1) fraction was observed for all metals, additionally for Cu, Pb, Zn in exchangeable fraction (F2) and in carbonate bound (F3) fraction for Cd and Zn. These results support the view that changes in the LF content may play a key role in controlling trace metals mobility, especially in long-term contaminated soil.

## Introduction

An increasing world population and economic development lead to accumulation of heavy metals in the soil at elevated levels, and disturb natural processes occurring in the environment [1]. Many authors emphasise that soil organic matter (SOM) has a strong influence over the fate of heavy metals in polluted soil; nonetheless the role depends on its amount, composition and dynamics [2, 3]. Over the last few decades much attention has been paid towards various organic matter fractions and their influence on trace metal mobility in soil. Kabata–Pendias & Pendias [4] and others [5, 6] indicated that heavy metals bound onto insoluble humic substances are relatively immobile, while the water soluble fraction of organic matter may increase metal mobility and bioavailability to biota. Recently, however, the usefulness of chemical fractions of organic matter have become increasingly criticized because of the technical limitations such as non-selectivity of chemical reactants and, as well, these pools are not clearly related to the dynamics of SOM [7–10].

Another approach for studying the relation between heavy metals and soil constituents is physical fractionation. This technique separates soil organic matter on the basis of particle size and density. The two most commonly isolated forms of physical uncomplexed organic matter are light fraction (LF), separated using liquids of specific density (1.6–2.0 g·cm^-3^) [10–12], and particulate organic matter (POM) sequestered by size (>53μm) [3, 10, 13]. These fractions are highly susceptible to breakdown when ecosystems are disturbed and they turn over much more quickly than organic matter bound to mineral phase [12]. That’s why physical fractions have been successfully used in research concerning organic matter dynamics. Recently, POM has been used to analyse the distribution of trace elements in the soil components. Studies carried out on metal contaminated soils by Balabane *et al*. [14], Balabane & van Oort [15], Dumat et al. [2], Labanowski *et al*. [13], Wiatrowska et al. [16] and Zhou *et al*. [3] have shown that this fraction of organic matter (POM, size-fraction) is metal-enriched compared to other soil constituents. Concerning the light fraction there have only been few research projects about metal association with this pool of organic matter.

If the light fraction occurs to be metal-enriched, mineralization of this fraction may lead to remobilization of attached elements. As climate change is assumed to alter to a certain extent soil organic matter quantity and quality, and most of the forecasts for Poland have assumed increasing yearly mean temperatures and decreasing amounts of precipitation during the summer period (increasing water deficiency in soil), this projected weather condition will favour accelerated mineralization of SOM [17]. According to Kirschbaum [18] increasing global temperature will result in releasing large amount of CO_2_ from soil, and this process will be more visible in cooler regions than in temperate. Taking into consideration that susceptibility of light fraction for oxidation processes is a few times higher than POM [9, 10, 11, 19], improving our knowledge about the interaction between light fraction and heavy metals is essential.

The purpose of this paper was to assess the role of light fraction in Cd, Cu, Pb and Zn binding in contaminated soil by metallurgical fallout. Furthermore the effect of turnover of light fraction on heavy metal mobility was also studied in order to estimate the potential risk linked with it. This study should enhance understanding of the role of LF in metals which have a strong influence on the efficiency of the remediation process of soil polluted with trace elements.

## Materials and methods

### Site, soil and experimental setup

**Fig 1:**
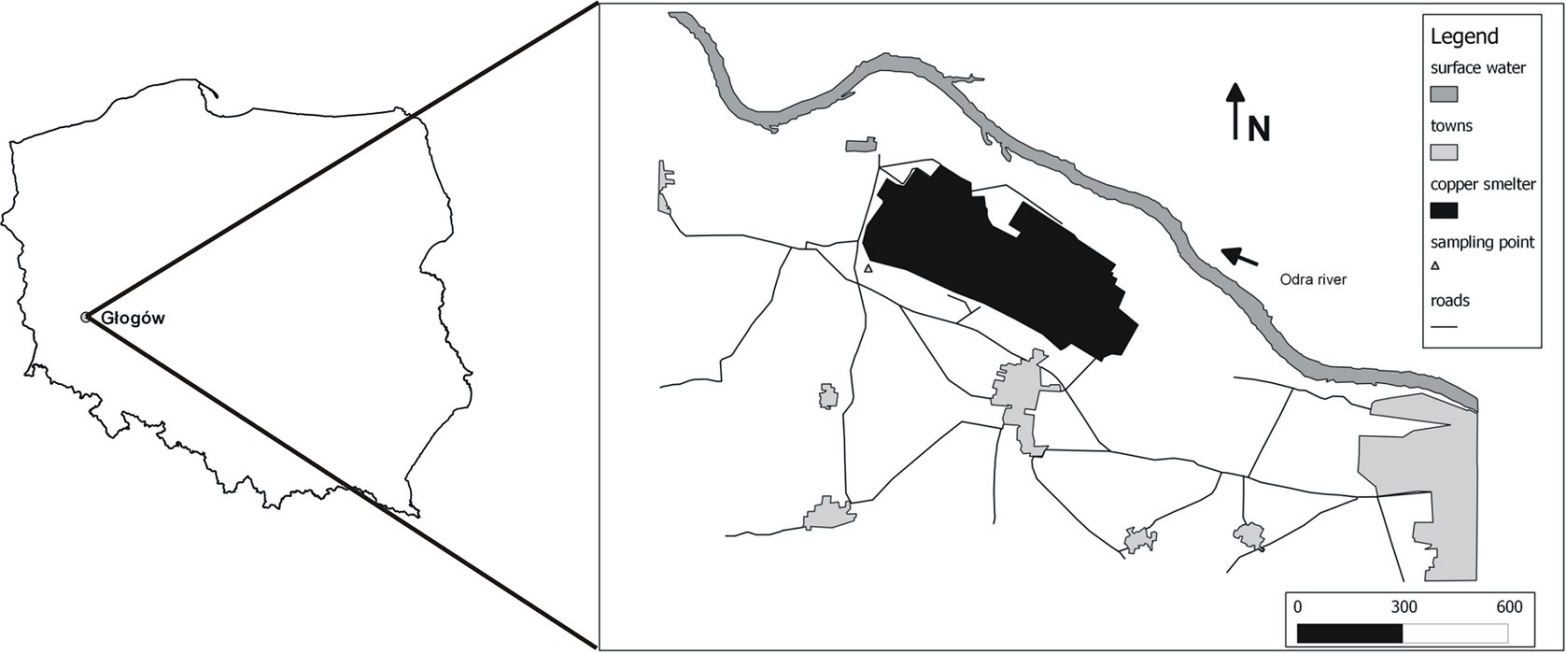
Localization of sampling point.

The incubation experiment was conducted on surface soil horizon sampled from the previously-existing sanitary zone (an area excluded from agricultural cultivation) of the copper smelters in Głogów in Western Poland (51^0^ 41.157 N; 15^0^ 57.276 E) (Fig 1). The region has a temperate climate with a mean annual temperature of 8.6°C and a precipitation of 549 mm. The Głogów smelters I and II began production in 1971 and 1974 respectively and they have been emitting into the atmosphere huge amounts of dust containing large amounts of trace elements; mainly Cu, Pb, Zn, Cd and As [20]. The peak period of dust emission was recorded in 1974–1979 reaching 14 x 10^3^ Mg per year and at present it has decreased to 179 Mg per year. In the period of activity between 1971 and 1990 no modern filtering systems were used and dust particles emitted ranged from a few to several tens of micrometers. The sanitary zone around Głogów smelters was designated in 1990 and occupied 2840 ha; this land was afforested with black poplar (*Populus nigra L*.) and Canadian poplar (*Populus x canadensis* Moench var. *serotina* (Hartig)). At the end of 2005, the sanitary zone was abolished by administrative decision, as a result of the introduction of modern technologies limiting pollutant emissions [21].

In this research project we focused on a surface layer (0–10 cm) strongly affected by atmospheric deposition of smelter fallout. Bulk sample of several kilograms of the loamy-sand textured soil (Brunic Arenosol, [22]) was collected. Prior to the incubation study, the soil was passed through a 4-mm sieve and water was added to 50–60% of its water holding capacity. Then soil was mixed with either pig manure (at the dose of 40 Mg ha^-1^) or triticate straw (at a dose of 4.5 Mg ha^-1^). In addition, the authors left some of the soil unamended (no organic material added). This gave three levels of organic amendment, which were denoted ‘manure’, ‘straw’ and ‘nil’. Each of six experimental combination were run in three replicates. The combinations were incubated at room temperature (20–22°C) for 450 days, during which soil moisture was monitored and water losses were compensated by irrigation. Soil samples for the physico-chemical and chemical analyses were sampled after 14 and 450 days of incubation. Soil samples taken after 14 days of incubation were treated as a reference level and all analyses were compared to this sampling time.

### Laboratory methods and analyses

All soil analyses were conducted on air-dried samples, which were passed through a 2 mm sieve. Density fractionation of soil samples was achieved following Gregorich & Ellert [11]. Firstly, 25 g soil was dispersed in 50 ml sodium iodide (ρ = 1.7 g cm^-3^) for 60 minutes in a horizontal shaker and then centrifuged at 10000 rpm. The supernatant was filtered through fiberglass under suction. This process was repeated twice in order to separate the light fraction fully. After that, to remove sodium iodide from the light fraction, the material separated was flushed with 0.01 M calcium chloride followed by distilled water. Before further analysis the light fraction was dried (60°C for 48 h).

Organic carbon in the bulk soil and the light fraction was determinated by dry combustion method (Multi N/C 3100 Analytikjena). As the soil contained calcium carbonate, samples were treated with 10% HCl before analysis. Dissolved organic carbon (DOC) was analyzed according to PN-E 1484 [23] protocol on a Multi N/C 3100 analyzer. Total nitrogen was determined by the Multi N/C 3100 analyzer (Analytikjena) after acid mineralization in the presence of a K_2_SO_4_ and CuSO_4_ mixture. The instrument detection limits for DOC, N and C_org_ in the solid samples were 4 μg·dm^-3^, 50 μg·dm^-3^ and 30 μg·kg^-3^ respectively. Soil pH was measured in a 1:1 (w/v) ratio of soil and water suspension. The cation exchange capacity (CEC) was determined by a modified Mehlich method as the sum of extractable bases with a mixture of BaCl_2_-triethanolamine and extractable acidity with sodium acetate [24].

In order to assess the heavy metal pools, the following single extractants were used: *aqua regia* (sample:*aqua regia* 1:10) for ‘total’ content in the bulk soil and the light fraction samples; 0.05 M EDTA [25] in a 1:20 ratio of light fraction:solution to extract potentially mobile forms from the light fraction of organic matter. In addition, sequential analysis was introduced to show changes in metal binding during incubation, when forced mineralization processes occurred.

The sequential analysis protocol applied in this research project was a modified Tessier’s method [26]. It was designed to separate metals into six operationally defined fractions: reversibly physically sorbed (F1) water extractable; exchangeable—weakly sorbed (F2), specific sorbed and carbonated bound (F3), metals associated mainly on iron and manganese oxides (F4), strongly complexed by organic matter (F5) and residual (F6). In this protocol one gram of soil was weighted and placed in a glass 100 ml centrifuge tube. The following extractions were made sequentially:

(F1) sample extracted with 10 ml of deionized water for 8 h at 20°C on an orbital shaker.
(F2) the residue from (F1) extracted with 15 ml of 1 M MgCl_2_, pH 7 for 8 h at 20°C on an orbital shaker.
(F3) the residue from (F2) extracted with 15 ml of 1 M CH_3_COONa, pH 5 for 8 h at 20°C on an orbital shaker.
(F4) the residue from (F3) extracted with 20 ml of 0.04 M NH_2_OH·HCl in 25% CH_3_COOH for 8 h at 96°C (water bath).
(F5) the residue from (F4) extracted with 3 ml of 0.02 M HNO_3_ and 6 ml H_2_O_2_ at 85°C until all liquid evaporated. After cooling, 10 ml of 3.2 M CH_3_COONH_4_ in 20% HNO_3_ was added, sample was shaken for 1 h at 20°C on an orbital shaker.
(F6) the residue from (F5) was digested for 24 h with 40% HF then 2 ml of 0.65 M H_3_BO_3_ was added and shaken for 2 h, and finally diluted to 10 ml with deionized water.

After each extraction, the supernatant was separated by centrifugation for 10 minutes at 6000 rpm. Concentrations of Cd, Cu, Pb and Zn were measured by flame atomic absorption spectrophotometry (FAAS), with detection limits of 0.0015 mg·dm^-3^ for Cd, 0.0013 mg·dm^-3^ for Cu, 0.0023 mg·dm^-3^ for Pb and 0.009 mg·dm^-3^ for Zn.

All soil analyses were done in duplicate for each soil sample studied, with mean values reported and the precision was given as standard deviation. The values of the relative standard deviation (RSD) for LF mass did not exceed 8.94%, for DOC 9.75% and 6.88% for all metals.

### Statistical analysis

Data are expressed as a mean ± SD of sixth determinations (each sample were analized in duplicate and each combination was incubated in triplicate). Student’s t-test followed by the least significant difference post hoc test was used to test the differences among combinations and incubation time. Additionally, one-way Anova (p < 0.05) and principal component analysis (PCA) were applied. Statistical analyses was done using the Statistica ver. 7.0.

## Results and discussion

### The general characteristic of the soil

Selected properties and trace elements content of the bulk soil (< 2mm) are reported in Table 1. The results are presented as the mean of the two replicates. Trace elements concentrations in the bulk soil were significantly higher than the local background level. The values for Cd, Cu, Pb and Zn content were in some cases higher than the legal Polish limit for post-industrial areas [27] (10, 300, 500 and 1000 ppm for Cd, Cu, Pb and Zn, respectively). Based on their abundance, soil was classified as heavily contaminated by Cu, strongly contaminated by Pb and contaminated by Cd and Zn [28]. High content of Cu and Pb in comparison to other metals in this soil was a result of emission of metallurgical dust, which contained mostly these elements.

**Table 1:** Selected physical and physicochemical properties and total trace element content of soil and organic amendments.

Variable	Contaminated soil	Pig manure	Triticale straw
OC / g · kg^-1^	59.6 ± 0.2	310 ± 3	456 ± 5
TN / g · kg^-1^	3.37 ± 0.08	15.3 ± 0.6	7.5 ± 0.2
C/N	17.7	20.2	60.8
pHwater	6.9 ± 0.04	-	-
CaCO_3_ / %	0.73 ± 0.02	-	-
CEC / cmol_c_ kg^-1^	14.39	-	-
Sand / %	82	-	-
Silt / %	17	-	-
Clay / %	1	-	-
Cd / mg · kg^-1^	4.06 ± 0.08	0.181 ± 0.003	0.093 ± 0.002
Cu / mg · kg^-1^	2500 ± 114	248 ± 7	338 ± 3
Pb / mg · kg^-1^	1267 ± 58	111 ± 4	70.2 ± 1.5
Zn / mg · kg^-1^	414 ± 17	1535 ± 36	288 ± 5

Data expressed as mean ± SD, n = 2

The soil had neutral pH (6.9), organic carbon content 5.96% and CEC 14.4 cmol_(+)_·kg^-1^. Such a high pH was a result of incorporation in the past of a huge quantity of CaCO_3_ to decrease trace element mobility. Soil texture was classified as loamy sand and it contained only 1% of clay fraction (Table 1). According to Oades [29], Paul et al. [30], Muller and Hoper [31] the ability of soil to protect organic carbon compounds against microbial decomposition depends on its clay content. Therefore, in this soil, protection of SOM would be low and could easily turnover releasing elements attached to organic compounds.

### Light fraction

Physical fractionation of soil organic matter (SOM) conducted on soil samples collected during the incubation experiment allowed to a pool of SOM with a density below 1.7 g·cm^-3^ to separate, which was further analysed according to its ability to bind trace elements. Amounts of the light fraction (LF) separated after 14 days of incubation were similar to a range reported by Gregorich and Ellert [11] for soil under forest (3–10%) and average around 6.7%. Independently to a type of organic material applied to the soil, a significant (at a level 0.005) increase in a share of LF was observed. This effect was more visible in the combination with manure, the mass of LF was higher (about 33%) in comparison to ‘nil’ combination (Table 2). In all combinations studied, LF occurred to be enriched in organic carbon, the content of C in this pool of organic matter was six-times higher than in the bulk soil. Despite low content observed for LF, the contribution of this fraction to total organic carbon content was substantial. Carbon in this fraction of SOM accounted 33% - 45% of soil C. These results were consistent with the data presented by Gregorich et al. [10], which showed that LF-C in forest soils accounted 8–74% of soil C. During the incubation period, when mineralization conditions prevailed, the share of LF significantly decreased (p < 0.05) and, additionally the effect of organic materials application was not visible any more (Table 2). Even though the mass of LF in soil diminished, at the end of the incubation time the contribution of this pool of SOM in soil-C stock was high and accounted for more than one-fourth (23–34%).

**Table 2:** Some characteristics of the light fraction separated from the combinations studied.

	Mass of light fraction		Organic carbon		
Experimental combination	14^th^ day / %	450^th^ day/ %	14^th^ day/g kg^-1^	450^th^ day/g kg^-1^	C:N final
Manure (M)	7.29 ± 0.49	4.39 ± 0.41	373.1 ± 33.4	275.1 ± 28.7	18.4
Straw (S)	7.18 ± 0.21	4.92 ± 0.35	378.5 ± 30.2	284.3 ± 2.3	19.4
Nil (N)	5.49 ± 0.33	4.80 ± 0.20	364.9 ± 30.1	254.4 ± 8.0	16.0

Data expressed as mean ± SD, n = 6

The Anova analysis (p < 0.05) confirmed that application of foreign organic materials has increased the LF mass. At the same time, the incubation time also influenced the content of the LF. With incubation time, the content of this fraction of soil organic matter decreased, with much larger changes noted in combination with manure and straw than nil. Faster breakdown of foreign organic matter may result from its greater susceptibility to decomposition and lack of inhibitory effect on soil microorganisms of trace elements.

### Metals content in the light fraction

**Fig 2:**
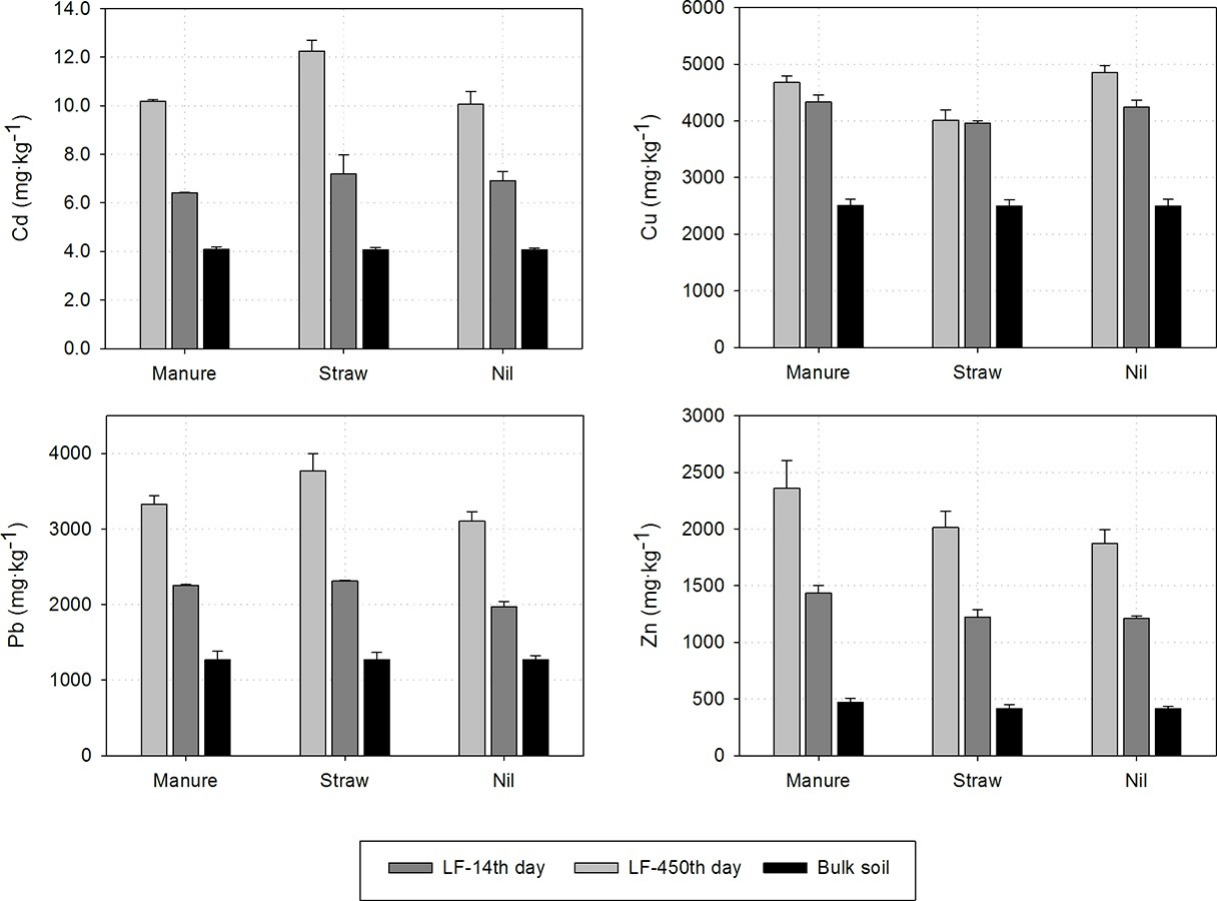
The stock of trace element in the bulk soil and the light fraction.

Regardless of the element considered each time the light fraction was always enriched between two to five times more in comparison to the bulk soil (Table 3, Fig 2). Similar trends were presented by Balabane et al. [14], Labanowski et al. [13], Wiatrowska et al. [16] and Zhou et al. [3], who studied metal enrichment of particulate organic matter (POM). Such a high metal content in this pool of SOM might be a result of higher content of organic C in LF fraction and probably a larger presence of reactive site on organic structure. However, the effect of initial input of metal-enriched residue (precursors of LF) can not be excluded. Taking into account the content of trace elements in LF, relative mass of this pool of organic matter in soil caused LF-metal associations in general to represent an important contribution to total metal concentration of the bulk soil samples. These metal associations with LF at the beginning of incubation accounted for 16.2%, 11.9%, 18% and 32.3% of total Cd, Cu, Pb and Zn stock, respectively. Even though Cu and Pb exhibit stronger affinity to SOM [32–33] than the rest of element studied, it was not reflected in the enrichment of the light fraction. The highest enrichment was observed for Zn then Pb, Cd and the lowest for Cu. These results were consistent with data presented by Wiatrowska et al. [16], who also observed a stronger tendency of Zn to bind with POM fraction than Pb.

**Table 3:** Total amount of Cd, Cu, Pb and Zn (in mg kg of fraction^-1^) in the light fraction (n = 6).

	Total content / mg kg^-1^							
	14^th^ day of incubation				450^th^ day of incubation			
Experimental combination	Cd	Cu	Pb	Zn	Cd	Cu	Pb	Zn
Manure (M)	10.19 ± 0.06	4680 ± 116	3326 ± 115	2358 ± 248	6.41 ± 0.02	4334 ± 119	2255 ± 10	1434 ± 70
Straw (S)	12.24 ± 0.46	4011 ± 188	3764 ± 235	2013 ± 142	7.19 ± 0.78	3959 ± 45	2311 ± 12	1220 ± 68
Nil (N)	10.06 ± 0.54	4860 ± 118	3104 ± 3	1873 ± 10	6.92 ± 0.37	4241 ± 129	1973 ± 66	1210 ± 20

Application of organic materials to the contaminated soil increased trace element content in the LF. Only Cu with the application of straw resulted in a slightly lower content in LF. This effect was observed for both sampling periods. This soil enrichment with organic materials did not influence the share of trace elements associated with LF. Also Mohamed et al. [34] informed that after soil amelioration with organic materials (rice straw, pig manure and green manure) increased Cu and Cd in POM fraction was observed. Additionally, the amounts of metals associated with POM increasing with the dose of organic materials.

### Extractability of trace elements in light fraction

Several studies considered that EDTA-extractable trace elements were easily uptaken by the plant root system [35–36]. Therefore, this pool of heavy metals is considered as potentially bioavailable, even though no studies have reported that plants can directly utilize the metal associated with LF. Independently of the combinations studied, the amounts of trace elements extracted with EDTA were high (Table 4). In the case of Cd, Pb and Zn concentrations were even higher than the bulk soil, in particular, for zinc, whose content was even three times higher. The percentages of EDTA-extractable Cd, Cu, Pb and Zn were 45.1–49.5%, 58.4 0–75.1%, 53.6–65.3% and 51.3–68.9%, respectively. Application of organic materials did not greatly change the share of EDTA-extractable pool of heavy metals, except combination with triticale straw that showed significant lower share of potentially bioavailable pool of Cu and Pb.

**Table 4:** EDTA-extracted content of Cd, Cu, Pb and Zn (in mg kg of fraction^-1^) in the light fraction.

	Cd	Cu	Pb	Zn
	mg·kg^-1^			
Experimental combination	0.05M EDTA	0.05M EDTA	0.05M EDTA	0.05M EDTA
14^th^ day of incubation				
Manure (M)	5.04 ± 0.18	2078 ± 60	2147± 69	1210 ± 41
Straw (S)	5.52 ± 0.25	2197 ± 79	2016± 69	1387 ± 57
Nil (N)	4.59 ± 0.23	2331 ± 93	2027± 65	1231 ± 36
450^th^ day of incubation				
Manure (M)	3.87 ± 0.19	1916 ± 63	1442 ± 71	1001 ± 46
Straw (S)	5.27 ± 0.22	1992 ± 58	1529 ± 43	839 ± 29
Nil (N)	3.91 ± 0.20	1628 ± 112	1417 ± 27	993 ± 41

### Changes in LF and trace element content

After 450 days of incubation, during which no fresh organic matter was introduced and the soil was maintained at 20–20°C, at the state of 50–60% of water holding capacity, a significant loss of light fraction was observed (Table 2). The loss of this pool of soil organic matter varied from 31.4% to 39.8% in combination with organic amendments. In the case of nil combination, the reduction was lower and amounted to only 12.6%. Such a response to the incubation time was a result of a combination of biotic (microbial activity) and abiotic (increased oxygen supply, high temperature) factors which favour mineralization processes of organic matter. As the light fraction is a short-term reservoir of plant nutrients and serves as a readily decomposable substrate for soil microorganisms, its amount is a balance between debris inputs and decomposition processes [12, 37]. In this research project fresh residue input was completely excluded and this led to a heavy decrease in LF share. Additionally, according to Song et. al [37] increased soil humidity may directly enhance microbes activity and accelerate soil carbon decomposition. A similar effect was observed by Liu et. al [38] with soil temperature. A significantly greater reduction of LF in combination with organic amendments in comparison to nil combination (Table 2), results from the introduction of fresh organic matter, susceptible to microbial decomposition, as it does not contain inhibitory level of trace elements.

Together with LF mineralization a decrease in the trace elements content in this pool of organic matter was observed. In the case of Cd, Pb and Zn, the amounts extracted by *aqua regia* mixture were lower by about 40% in comparison to the initial level. Only for Cu a small decreased was observed, by 1.3–12.7% (Table 3). This might be a result of either preferential decomposition of low Cu-contaminated LF or strong sorption of this metal by organic functional groups or both. However, even after 450 days of incubation the light fraction was, in all combinations, more enriched with trace elements in comparison to the bulk soil. A similar trend was also noted for EDTA-extracted metals. The amounts of bioavailable pool of trace elements associated with LF decreased during incubation time, but were still high and for Pb and Zn were even higher than the bulk soil.

### Sequential analysis

**Fig 3:**
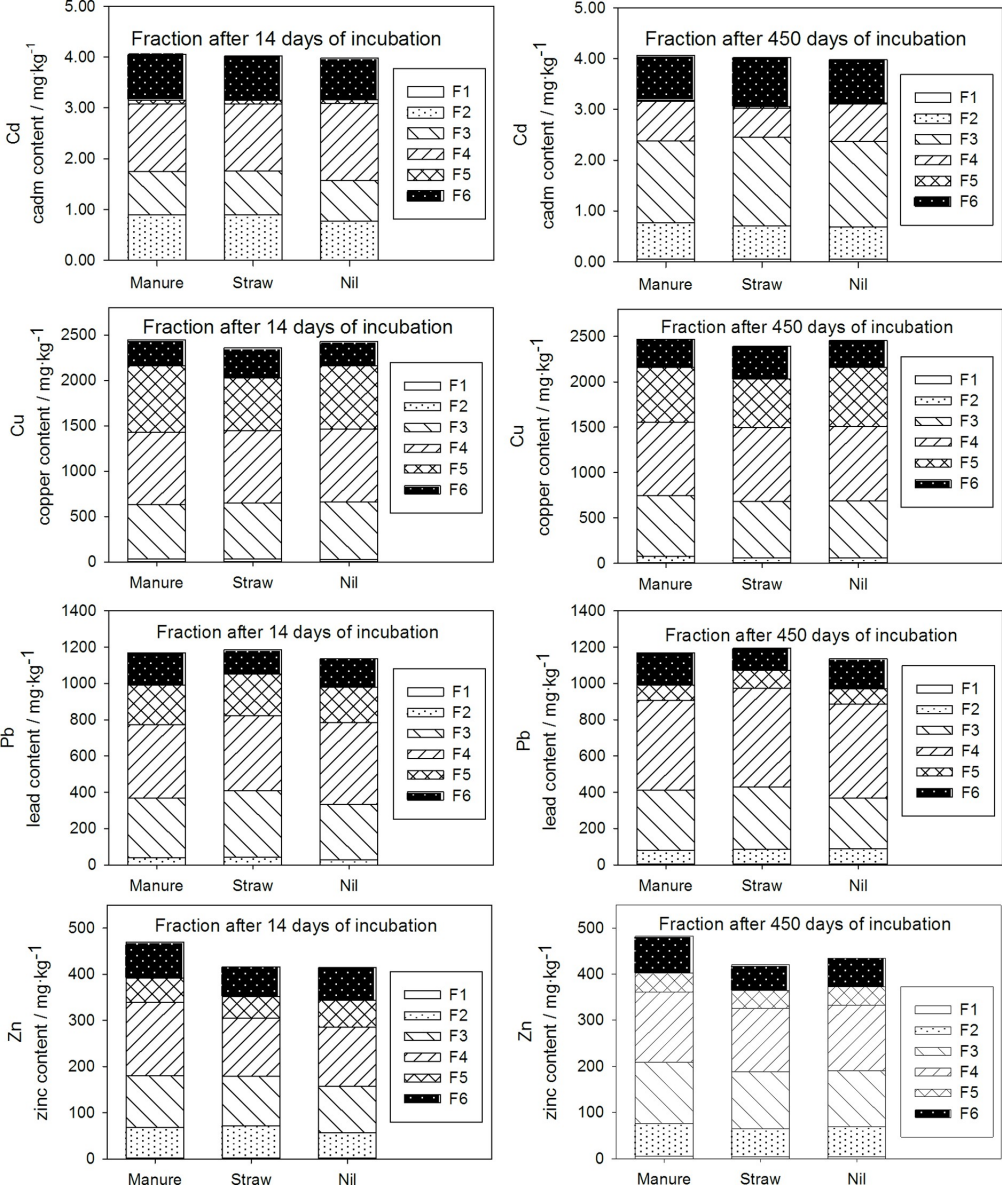
Metal speciation in particular combinations. Fractions: F1 = water soluble, F2 = exchangeable, F3 = bound to carbonates, F4 = bound to Fe-Mn oxides, F5 = bound to organic matter and F6 = residual.

In order to show the changes in the forms of trace elements in soil after mineralization of LF, a sequential analysis was carried out. Six fractions were identified in the analysis, but most attention was focused on potentially mobile fractions (F1, F2 and F3). The distribution pattern among these fractions varied slightly between trace elements. However, regardless of the element studied, the largest share was observed in fraction IV; bound to manganese, aluminium and ferrous oxides (Fig 3), except Cd after 450 days of incubation. The dominance of an oxide-occluded fraction in contaminated soil has been reported by other investigators [39–41]. Despite the high total content of metals (Table 1), the water-soluble fraction (F1) was negligible in all combinations and did not exceed 0.5%. The lowest concentration in the water-soluble fraction was seen for Pb. This was related to its high electronegativity and softness (according to Pearson acid-base theory) [42–43]. Despite the reported affinity of Pb to soil organic matter [4], the organic bound fraction (F5) was relatively small and did not exceed 20% of total Pb in soil.

**Fig 4:**
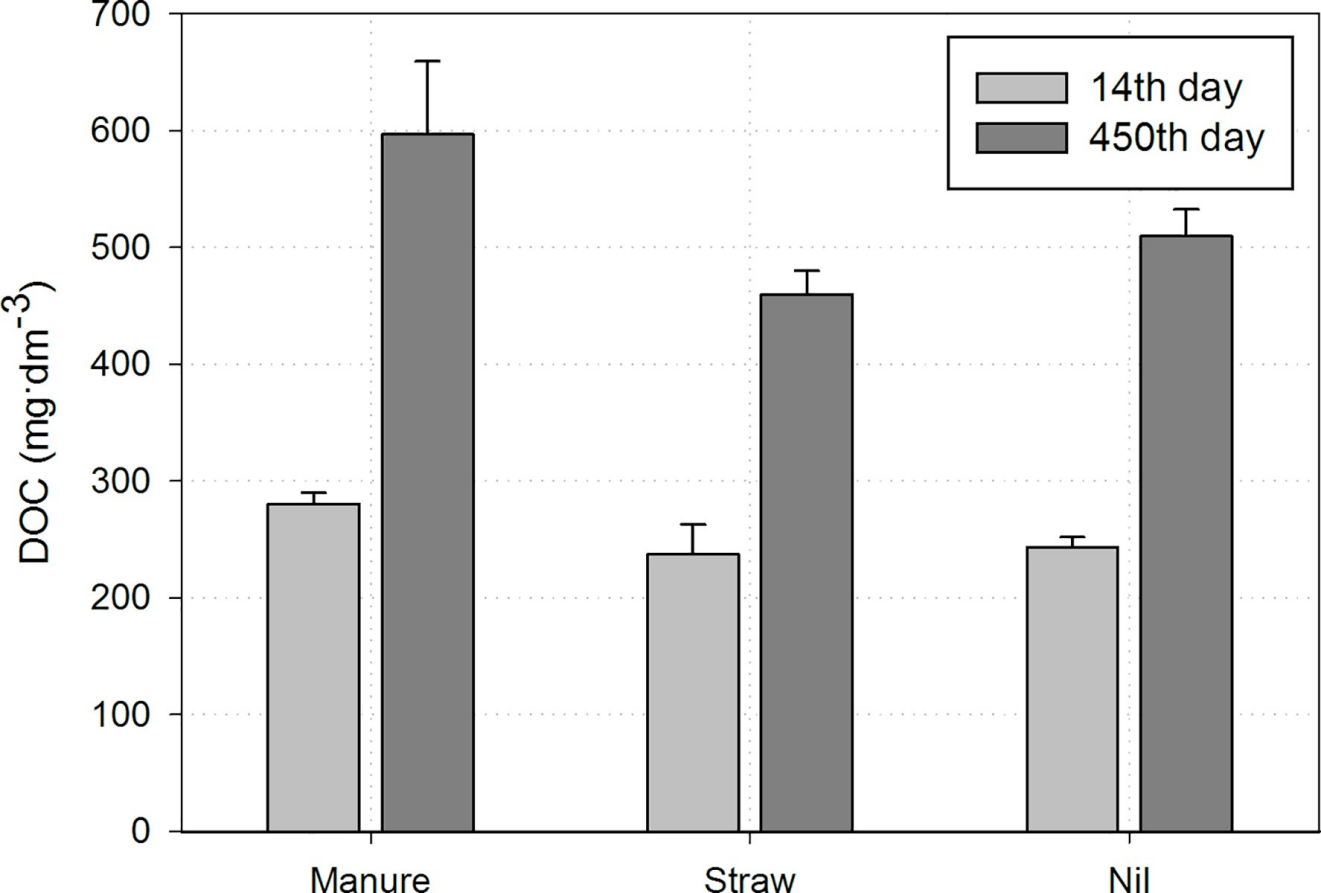
Changes in DOC concentration during the incubation time.

After 450 days of incubation, the metals distribution among the separated fractions have changed slightly. As a result of the mineralization of the LF, the amounts of metal associated with organic matter (F5) has decreased. At the same time, an increase in the proportion of metals in the water-soluble fraction (F1) for all metals was noted (Fig 3). The most visible change in this fraction (F1) was noted for Cd, whose concentration increased by 6 and 7 times in manure amended soil and in other combinations, respectively. Moreover, for Cu, Pb and Zn an increase of F2 share was observed and for Cd and Zn of F3. The change in F2 and F3 fractions were probably connected with adsorption of released metals by soil colloids. Additionally, neutral soil pH and the presence of carbonates might create conditions for Zn and Cd carbonate precipitation [44]. Higher trace elements mobility (F1) could be related to dissolved organic carbon (DOC) concentrations, which doubled during the incubation period (Fig 4). Many authors have documented the fact that the dissolved organic matter increases metal mobility and uptake by biota [5, 45–47]. The organic acid presented in the DOC can act as an chelating agent and therefore enhance the mobilization of metals. Hu et al. [48] noticed that the effect of DOC on trace metal mobility varies depending on its type. Fresh dissolved organic carbon had a more noticeable effect on Cu sorption than degraded dissolved organic carbon. This could partially explain higher amounts of trace elements extracted from LF in combination with straw than with manure. However, it is well established that organic carbon compounds show higher affinity toward Cu and Pb [5, 47]. In the present study, no difference in Cu content in water-soluble fraction was observed, even when the DOC concentration doubled. Du et al. [49–50] reported that Pb has a stronger affinity towards soil colloids, both mineral and organic, than Cu. Such behaviour could be explained by soil pH and interaction among trace elements, accompanying ions and soil colloids. Most soils are characterized as slightly acidic or acidic. In such conditions a stronger affinity towards the sorption sites is observed for Pb. According to the results of Komisarek and Wiatrowska [51] and Wiatrowska et al. [52], Cu was preferentially sorbed by soil with neutral and alkaline pH. At the same time lead ions (PbOH^+^) compete with Ca^2+^ for sorption sites, as they have similar ionic radius. Taking into account the pH (6.9), where competition between Pb and Ca was being observed, Cu^2+^ showed the strongest affinity to soil colloids among the trace elements studied. Nonetheless, a preferential mineralization of low contaminated debris cannot be excluded.

**Fig 5:**
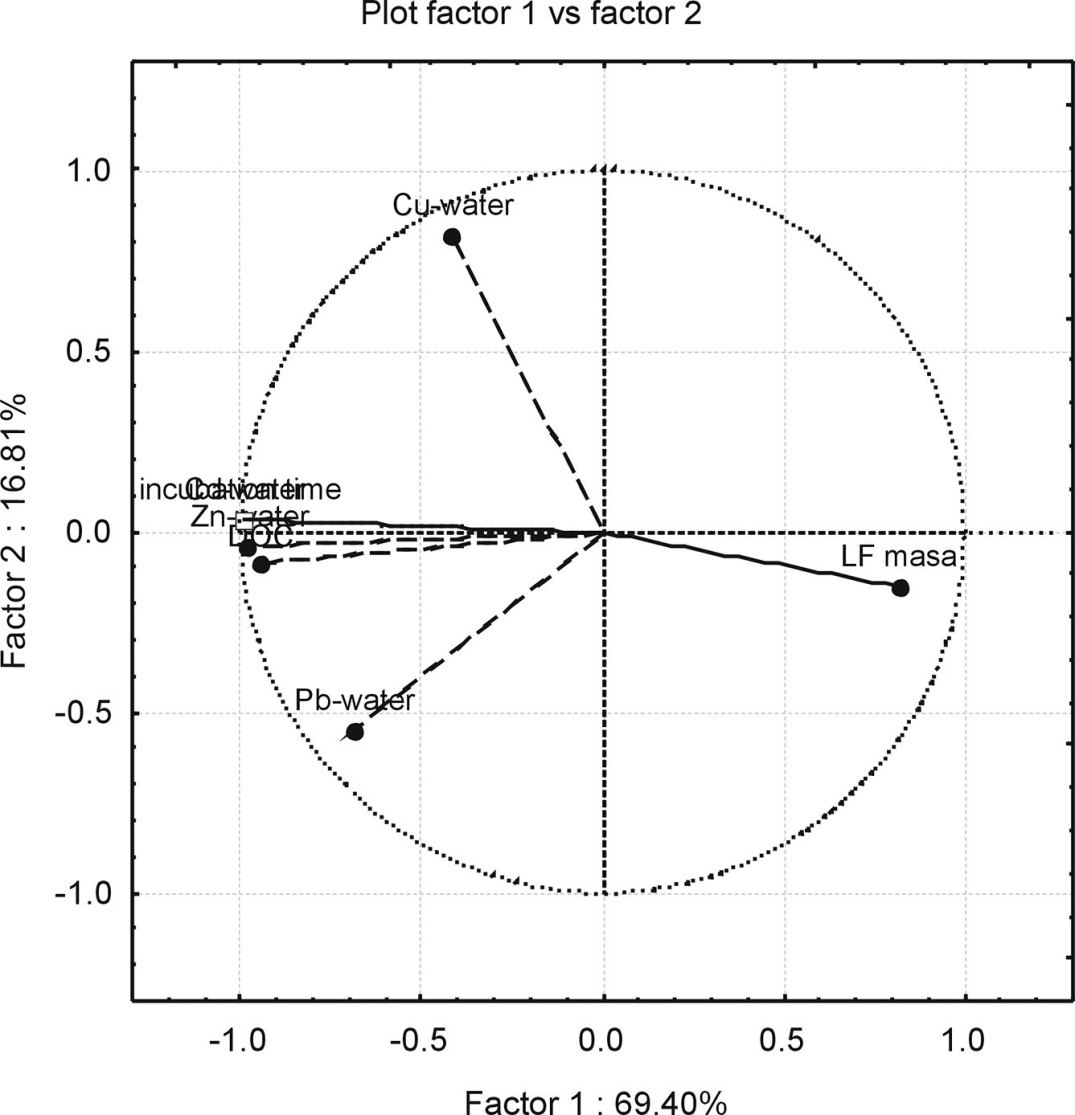
Graph of a principal component analysis.

To identified factors which control the concentration of water-soluble form of trace elements in soil, correlation analysis and principal component analysis (PCA) were applied. The statistic analyses showed that both LF content and DOC concentration were the main factors affecting distribution of metals in F1 (watersoluble fraction). The LF mass was strongly negatively correlated with F1 of Zn and Cd and in a lesser extent with Pb and Cu. In turn, a positive correlation was found with DOC and Cd, Pb, Zn concentrations. The result of PCA is presented as a diagram (Fig 5). The statistic analyses confirmed that LF could be a significant source of trace elements. As a results of mineralization of the LF, the DOC concentration increases and easily mobile association with trace elements are created. In this study, Cd, Zn and Pb reacted the most to increases in DOC concentration.

## Conclusions

Although the mass of LF was low in the bulk soil (5.49%) and it increased slightly after addition of organic amendments (7.18–7.29%), it was observed that Cd, Cu, Pb and Zn stock reached 16.2%, 11.9%, 18.0%, 32.3%, respectively. An application of organic materials increased metals content in LF, except for Cu in combination with straw. In addition, trace elements association with LF showed a high level of EDTA extractability, indicating their potentially high bioavailability. After 450 days of incubation, the content of the LF decreased. It is a pool of soil organic matter that reacts quickly to external factors. Therefore, a reduction of LF was observed at the level of 31.4–39.8% for combinations with organic amendments. In the nil combination the LF decreased by only about 12.6%. At the end of the experiment, LF accounted on average for 7.3%, 7.8%, 8.1% and 14.0% stock of Cd, Cu, Pb and Zn, respectively. This fraction of soil organic matter not only represents a significant contribution to total metal content, but also becomes an important potential source of re-releasing active forms of trace elements. Mineralization of LF changed metal distribution, increased metals content in the water-soluble, exchangeable and carbonate-bound fractions as the pool of trace elements bound to organic matter decreased. However, the strongest increase in metal mobility was observed for Cd and Pb, despite the fact that lead and copper were characterized by the largest share in F5 fraction.

Results obtained showed a significant reduction in LF content when the ecosystem is disturbed. Therefore, the study of interactions between metals and LF fraction will be very useful in terms of better management of long-term contaminated soil, especially under the conditions of the climatic changes forecasted.

## Supporting information

S1 File The stock of trace element in the light fraction. (XLS)

S2 File Metal speciation in particular combinations. (XLS)

S3 File Changes in DOC concentration during the incubation time. (XLS)
